# Diagnosis and surgical treatment of chronic destructive septic hip arthritis

**DOI:** 10.1186/s42836-025-00305-2

**Published:** 2025-04-09

**Authors:** Zhishuo Zhang, Zida Huang, Xinyu Fang, Guochang Bai, Wenbo Li, Wenming Zhang, Chaofan Zhang

**Affiliations:** 1 Department of Orthopaedic Surgery, the First Affiliated Hospital, Fujian Medical University, Fuzhou, 350005 China; 2 Department of Orthopaedic Surgery, National Regional Medical Center, Binhai Campus of the First Affiliated Hospital, Fujian Medical University, Fuzhou, 350212 China; 3 Fujian Provincial Institute of Orthopedics, the First Affiliated Hospital, Fujian Medical University, Fuzhou, 350005 China; 4 Fujian Orthopaedic Bone and Joint Disease and Sports Rehabilitation Clinical Medical Research Center, Fuzhou, 350005 China

**Keywords:** Septic hip arthritis, Diagnosis, One-stage arthroplasty, Two-stage arthroplasty

## Abstract

Septic hip arthritis (SHA) is a relatively rare but hazardous disease. Much controversy exists regarding the definition, diagnosis and treatment of chronic destructive SHAs. This review aims to provide an overview of the diagnostic and therapeutic approaches for chronic, destructive SHA and suggest possible research directions for this disease’s future diagnosis and treatment. There is no unified naming or classification standard for SHAs. Chronic destructive SHA still requires a comprehensive diagnosis combining history, signs, bacterial culture, histopathological examination, inflammation and other indicators, of which metagenomic next-generation sequencing is a promising diagnostic tool. Previous treatment options for this disease include debridement, debridement + Girdlestone femoral head and neck resection, and debridement + Girdlestone femoral head and neck resection + two-stage arthroplasty. Among them, one-stage spacer implantation + two-stage arthroplasty is the current standard surgical option with a high success rate and low reinfection rate, while one-stage arthroplasty is a new treatment option proposed in recent years with unique advantages but limitations in terms of surgical indications. In the future, more high-quality studies are needed to provide the latest evidence to support clinical decision-making.

## Introduction

Septic hip arthritis (SHA) is a joint disease in which microorganisms invade the synovial membrane of joints and release various toxic substances, such as alpha haemolysin [1], resulting in purulent effusion in the joint capsule and destruction of the synovium [2]. It is a relatively rare but hazardous disease. If left undiagnosed and untreated, it can become chronic and persistent, resulting in progressive cartilage, bone destruction and, ultimately, loss of function [3]. The global incidence of SHA is estimated to be approximately 7.8 in 100,000 per year, but the mortality rate is approximately 10%, placing a heavy burden on society [4].

Currently, there are many controversies in the definition, diagnosis and treatment of chronic destructive SHA. Numerous studies have reported different surgical options combined with different therapeutic outcomes [5]. This article provides a review of the current status of treatment for chronic destructive SHAs. We focused on the treatment of chronic destructive SHAs, with an emphasis on hip debridement preservation and arthroplasty for chronic destructive SHAs.

## Definition of chronic destructive SHA

At present, there is no standardised nomenclature or classification for SHAs. For example, “septic” can be expressed as “suppurative” or “pyogenic”. The duration of infection can be categorised into acute SHA (< 3 weeks) and chronic SHA (> 3 weeks) [5]. The causes of infection can be categorised as primary or secondary. Primary SHA usually originates from bloodstream transmission of an infection elsewhere in the body, whereas secondary SHA usually originates from hip surgery or procedures that do not include periprosthetic joint infection (PJI) [6]. The nature of the infection can be categorised as destructive SHA, persistent SHA, quiescent SHA or cured SHA [7]. The term “chronic destructive” SHA refers more to the active phase, specifically to SHA associated with persistent infection by pathogenic microorganisms.

The purpose of categorising SHAs is to better guide treatment. For instance, acute and chronic SHA can have completely different treatment options. In addition, the wide variety of nomenclature used can easily lead to a biased understanding of the concept of SHA, making clinical diagnosis and treatment difficult. Heterogeneity among studies with different definitions and categorisation criteria also make the evaluation and comparison of studies difficult. Therefore, it is important to systematically name and categorise SHAs to guide clinicians better in diagnosis and treatment.

## Diagnosis of chronic destructive SHA

There are no standardised diagnostic criteria for chronic destructive SHA. A comprehensive diagnosis is still needed in conjunction with history, signs, bacterial culture, histopathological examination, inflammatory markers, etc. The manifestations associated with the early diagnosis of SHA have been summarized in Table 1. Clinical signs of SHA may manifest as sinus tracts communicating with the joints, localised redness, swelling, tenderness, exudation, joint pain and fever. Up to 90% of patients will have a low-grade fever [4, 8]. However, when a patient is immunosuppressed (e.g., with RA), the inflammatory response may be attenuated, resulting in a more ambiguous clinical picture[5].

**Table 1:** Manifestations associated with early diagnosis of SHA

Category	Specific manifestations
**Symptoms**	
Pain	Persistent hip joint pain, aggravated by activity
Limited mobility	Reduced range of motion in the hip joint, limited flexion, abduction and internal rotation
Fever	Low-grade or intermittent fever
Systemic symptoms	Fatigue, loss of appetite, weight loss, etc
**Signs**	
Swelling	Soft tissue swelling around the hip, possibly accompanied by redness and increased skin temperature
Tenderness	Significant tenderness in the anterior hip or groin area
Joint deformity	Flexion contracture (chronic infection)
Sinus	Skin sinuses accompanied by purulent discharge (advanced stages of chronic infection)
**Laboratory Tests**	
WBC	Elevated WBC and neutrophil percentage (active infection)
CRP and ESR	Significantly elevated
**Joint fluid analysis**	Purulent and significantly elevated WBC (usually > 50,000/mm^3^) Gram staining, positive culture and mNGS
**Imaging**	
X-ray	Joint space narrowing, bone destruction, osteoporosis, periarticular sclerosis and osteophyte (late stage)
CT	Bone destruction, sequestrum formation and periarticular abscesses
MRI	Joint effusion, synovial thickening, bone marrow edema and surrounding soft tissue infection

*CRP* C-reactive Protein, *CT* Computed Tomography, *ESR* Erythrocyte Sedimentation Rate, *MNGS* Metagenomic Next-generation Sequencing, *MRI* Magnetic Resonance Imaging, *SHA* Septic Hip Arthritis, *WBC* White Blood Cell Count

Inflammatory markers such as serum C-reactive protein (CRP) and the erythrocyte sedimentation rate (ESR) may be elevated in chronic SA but have low specificity. A joint fluid white blood cell count > 50,000 cells/mm^3^ or a polymorphonuclear leukocyte percentage > 90% (PMN%) is used as a common criterion for the diagnosis of SHA [9]. However, SF < 50,000 or PMN% < 90% does not necessarily rule out SHA, as elderly patients or immunocompromised patients may not produce such a strong response [5]. Recent studies have shown that the serum neutrophil-to-lymphocyte ratio (NLR) and synovial fluid NLR (SF-NLR) have superior diagnostic and prognostic power for SHA compared with current clinical criteria [9]. Synovial lactate is also a predictor of inflammation in SHAs, with synovial lactate levels > 10 mmol/L significantly increasing the incidence of SHAs [10].

Imaging methods effectively evaluate the condition of the joint and surrounding soft tissues. Radiography is preferred because it can show evidence of cartilage or subchondral bone destruction and effusion. Ultrasonography is also a safe and cost-effective method for evaluating effusions. Magnetic Resonance Imaging (MRI) not only has a high sensitivity and specificity for assessing the condition of cartilage, surrounding soft tissues and bone, but it can also detect osteomyelitis.

Gram staining, culture, cell counting and analysis by arthrocentesis remain the current “gold standard” methods for the diagnosis of SHA. It was reported a wide variation in the culture-positive rate of pathogen in SHA in the known literature, ranging from approximately 50%–100% [3, 6, 7, 11–20]. The most common pathogen is Staphylococcus aureus (30%–50%), including methicillin-sensitive Staphylococcus aureus (MSSA) and methicillin-resistant Staphylococcus aureus (MRSA). This is followed by coagulase-negative staphylococci (CoNS), Streptococcus spp, gram-negative bacteria, anaerobes, etc. [3, 6, 7, 11, 12, 14, 16, 17, 19–22]. However, empiric antibiotic treatment prior to arthrocentesis could result in low positive rates of staining and culture. I. K. Sigmund et al., proposed that three to six tissue specimens of the periprosthetic membrane and pseudocapsule should be collected at revision arthroplasty for PJI [23]. Given the similarity between PJI and SHA, it is crucial to specify the number of tissue specimens for histopathological analysis. With the development of molecular diagnostic technology, next-generation sequencing (NGS), especially metagenomic next-generation sequencing (mNGS), has been widely used in clinical diagnosis [24]. Studies have shown that mNGS technology has improved the detection rate of pathogenic microorganisms in infectious diseases such as oral, intracranial and lung infections [25]. In addition, some microorganisms that cannot be detected by routine culture, such as Rickettsia burgdorferi, Mycoplasma hominis and Mycoplasma salivarium, can be accurately identified by mNGS [26, 27]. Previous studies have also shown that mNGS can be used to effectively identify pathogens in the synovial fluid of patients with joint infections, especially for patients with negative cultures due to fastidious pathogens or recent use of antibiotics. This technique has demonstrated high sensitivity and specificity and is less affected by antimicrobial therapy [28–32]. Recent studies have also shown that mNGS can significantly improve the treatment success rate of SHA [7, 33]. Therefore, mNGS could be a promising diagnostic tool for chronic destructive SHA.

## Treatment of chronic destructive SHA

### Conservative treatment

For acute SHA, broad-spectrum intravenous antibiotics should be used at the beginning of treatment. After culture results are obtained, antibiotics that target the causative organism should be administered [3, 16, 19, 22, 34–36]. However, due to the persistent nature of chronic destructive SHA, it is usually difficult to kill pathogenic bacteria with antibiotic therapy alone. Therefore, for chronic destructive SHA, conservative treatment is a highly ineffective option and is indicated only for patients who cannot tolerate surgery.

In recent years, the literature has indicated that altering the release of platelets may be achieved by increasing the levels of inflammatory cytokines and/or chemokines and reducing the levels of anti-inflammatory cytokines. Such platelet alterations could confer a protective effect against bone and joint infections [37]. Additionally, it has been reported that ethylenediaminetetraacetic acid-normal saline irrigation with or without antibiotics is effective at eradicating S. aureus biofilm-associated infections both ex vivo and in vivo [38]. Vancomycin has been proposed as an infection prophylaxis drug for arthroplasty. To keep the administration period as short as possible, an initial target of 100% minimal inhibitory concentration (T > MIC) may be considered to ensure that the most resistant bacterial subpopulation is targeted. Some studies have shown that the time greater than the minimum inhibitory concentration (T > MIC) of vancomycin is greater than that of meropenem across all investigated compartments. Accordingly, treatment should be guided by local susceptibility patterns. This suggests that a more aggressive dosing approach than just choosing a broad-spectrum combination to achieve longer T > MIC in all the exposed tissues, including the application of local antibiotics, may be considered to lower the risk of acquiring an infection after arthroplasty [39]. These findings contribute to the further exploration of conservative treatment methods for chronic destructive SHA. However, these are all still in the realm of basic research.

### Debridement

#### Arthroscopic debridement

Previous studies have shown that acute SHA can be treated by either arthroscopic debridement or open debridement [5]. A systematic evaluation of arthroscopic debridement for the treatment of SHA by Darren de et al., revealed significant improvements in pain and joint function after the procedure [40]. Multiple studies have shown that arthroscopic debridement is a safe and effective method for treating SHAs [34, 35, 41]. Additionally, studies have shown that patients who undergo arthroscopic debridement for SA experience fewer surgical procedures, shorter hospital stays, lower visual analogue scale (VAS) pain scores and greater mobility than those who undergo open debridement [42, 43]. However, D’Angelo’s study [44] showed that arthroscopic debridement was not superior to open arthroplasty in the treatment of SHA. Lee et al. [34] also noted that although the infection recurrence rate with arthroscopic debridement for SA is lower than that with conservative treatment, multiple surgeries are often required to completely eradicate the infection. Stutz et al. [45] suggested that the effectiveness of arthroscopic debridement for treating SA is correlated with the stage of infection. In the initial stages of infection (acute phase), arthroscopic debridement results in better treatment outcomes and a better prognosis. Given the stubbornness and refractoriness of pathogens in chronic destructive SHA, arthroscopic debridement may not be suitable for eradicating infection in this disease [34]. Patients with articular cartilage and bone destruction or osteomyelitis who undergo arthroscopy or open debridement have a higher failure rate and may develop chronic SHA [16]. In conclusion, it is a viable, minimally invasive solution for patients with acute, undamaged SHA, but it is not effective in the treatment of chronic destructive SHA.

#### Open debridement

Most of the literature indicates that there is no significant difference in efficacy between arthroscopic debridement and open debridement for treating SA [4, 8]. However, some researchers still believe that compared with arthroscopic debridement, open debridement is more thorough. Studies have shown that open debridement can adequately drain SHAs and improve the functional outcome of joints [19, 46]. A study comparing arthroscopic debridement and open debridement revealed a greater failure rate of arthroscopic debridement when MRSA was the pathogenic factor, suggesting that open debridement should be considered if MRSA infection is suspected. Moreover, in cases where the joint surface is severely damaged, open debridement should also be performed to prepare for future arthroplasty or joint fusion [43].

However, similar to arthroscopic debridement, open debridement has been mainly applied to acute SHA in the past, with questionable efficacy in the treatment of chronic destructive SHA and limited reports in the literature. Moreover, open debridement is significantly associated with potential complications such as femoral head necrosis and dislocation [19, 34, 35, 46]. Therefore, the current mainstream view on treatment for chronic destructive SHAs still favours arthroplasty rather than debridement.

### Debridement + Girdlestone head and neck dissection

Girdlestone first described an approach in which arthroplasty was performed via complete resection of the proximal femur and debridement of the surrounding tissue. The approach achieved remarkable results in eradicating infection. However, the patient is immobile after the operation, leading to an increased risk of venous thrombosis in the lower extremities and possible sequelae such as limb shortening and joint contractures [47]. Therefore, this surgical method has been gradually eliminated and replaced by total hip arthroplasty (THA) after Girdlestone head and neck resection to improve hip function.

### Debridement + Girdlestone femoral head and neck resection + two-stage arthroplasty

Prior to the widespread use of spacers, the debridement + Girdlestone femoral head and neck resection + two-stage arthroplasty approach was the main surgical option for the treatment of SHAs. Chen et al. demonstrated that this surgical approach achieved satisfactory infection and eradication rates, but the complication rate was still unsatisfactory [20]. Several studies have shown that this option carries the risk of postoperative complications such as prosthesis dislocation, heterotopic ossification, haematoma and rotor nonunion [47, 48]. In addition, although the procedure reduces the patient's bed rest to approximately 3 months, there is still a risk of sequelae such as venous thrombosis of the lower extremities, limb shortening and joint contracture. Consequently, with the widespread use of the two-stage revision approach in PJI, this procedure has been gradually phased out.

### One-stage spacer therapy + two-stage arthroplasty

It has been documented that there is a ten-fold increased risk of PJI in patients with a history of septic arthritis who undergo THA compared with those who undergo THA for OA, with a ten-year cumulative incidence of 7%. The risk of any infection notably decreased as the time interval between the diagnosis of septic arthritis and THA increased [49]. Therefore, staged treatment with arthroplasty is critical for treating SA.

For PJI, the common surgical protocol is debridement + spacer implantation + two-stage total joint arthroplasty (TJA). Studies have shown that this protocol has achieved good efficacy in the treatment of PJI [50]. Considering the similarities between SA and PJI in terms of aetiology, diagnosis and treatment, the use of debridement + spacer implantation + two-stage THA in the treatment of chronic destructive SHA can also achieve a good prognosis. The use of a spacer avoids the risk of venous thrombosis and joint contracture in patients after Girdlestone femoral head and neck resection. In addition, two-stage arthroplasty ensures that the prosthesis is implanted in an infection-controlled state, thus reducing the risk of postoperative infection.

Several studies have shown that one-stage spacer implantation + two-stage arthroplasty has a high success rate in the treatment of SA (Table 2). The results of Sultan et al. [14] showed that this regimen overcame the high risk of thrombosis and sequelae after Girdlestone femoral head and neck resection and demonstrated the unique superiority of antibiotic-containing spacers. Bochatey et al. [51] showed that this regimen is suitable not only for quiescent SHAs but also for active infections. A study by Li et al. [52] showed that spacer implantation is uniquely superior for maintaining lower limb length and hip function.

**Table 2:** Studies of one-stage spacer therapy + two-stage arthroplasty for SA

Literature	Distribution of main pathogens	Total detection rate	Surgical Cases	THA/TKA	Average follow-up (months)	Success rate
Zhang et al. [7], 2022	MSSA 29.4% (5/17), MRSA 5.9% (1/17), CoNS 17.6% (3/17)	76.4% (13/17)	17	THA	36.7	100%
Li et al. [52], 2022	Staphylococcus aureus 20% (4/20)	35% (7/20)	11	THA	29.09	100%
Wei et al. [21], 2022	MSSA 17.1% (18/105), MRSA 13.3% (14/105), CoNS 6.7% (7/105)	59% (62/105)	55	THA	62	89%
Tan et al. [53], 2021	Staphylococcus aureus 24.2% (38/157)	67.5% (106/157)	110	THA&TKA	N/A	90.9%
Bochatey et al. [51], 2021	Staphylococcus aureus 50% (4/8)	100% (8/8)	8	THA	27	100%
Tan et al. [54], 2020	N/A	N/A	128	THA (93) &TKA (35)	62.4	86.7%
Russo et al. [11], 2021	MSSA 28% (7/25), MRSA 12% (3/25)	76% (19/25)	25	THA	85.2	96%
Kunze et al. [12], 2020	MSSA 14.3% (6/42), MRSA 9.4% (4/42), CoNS 23.8% (10/42)	73.8% (31/42)	12	THA	39.6	100%
Xu et al. [13], 2019	Staphylococcus aureus 10.9% (6/55), CoNS 27.3% (15/55)	69.1% (38/55)	55	THA	56.4	89%
Luo et al. [55], 2019	N/A	N/A	9	THA	24.2	100%
Sultan et al. [14], 2019	MSSA 25.8% (16/62), MRSA 4.8% (3/62)	61.3% (38/62)	15	THA	52.8	100%
Kao et al. [56], 2019	Staphylococcus aureus 3.9% (2/51)	21.6% (11/51)	51	THA	48.8	92.9%
Cho et al. [36], 2018	Staphylococcus aureus 40% (4/10)	60% (6/10)	10	THA	44.9	100%
Papanna et al. [22], 2018	MSSA 90.9% (10/11), MRSA 9.1% (1/11)	100% (11/11)	11	THA	70	100%
Anagnostakos et al. [15], 2016	Staphylococcus aureus 72.7% (16/22)	86.4% (19/22)	22	THA	44.8	87%
Fleck et al. [16], 2011	MSSA 42.9% (6/14), MRSA 14.3% (2/14)	78.6% (11/14)	14	THA	50	100%
Romanò et al. [17], 2011	MSSA 35% (7/20), MRSA 20% (4/20), CoNS 15% (3/20)	80% (16/20)	20	THA	56.6	95%
Huang et al. [3], 2010	MSSA 26.7% (4/15), MRSA 26.7% (4/15)	80% (12/15)	15	THA	42.5	100%
Bauer et al. [18], 2010	Staphylococcus aureus 40.9% (9/22), CoNS 27.3% (6/22)	100% (22/22)	13	THA	60	84.6%
Kelm et al. [19], 2009	MSSA 50% (5/10), MRSA 10% (1/10)	70% (7/10)	10	THA	12	87.5%
Diwanji et al. [6], 2008	MSSA 22.2% (2/9), MRSA 33.3% (3/9)	88.9% (8/9)	9	THA	42	88.9%
Chen et al. [20], 2008	MSSA 25% (7/28), MRSA 28.6% (8/28)	100% (28/28)	28	THA	77	86%

*CoNS* Coagulase-Negative Staphylococci, *MRSA* Methicillin-resistant Staphylococcus Aureus, *MSSA* Methicillin-sensitive Staphylococcus Aureus, *N/A* Not Available, *THA* Total Hip Arthroplasty, *TKA* Total Knee Arthroplasty, *SA* Septic Arthritis

PJI is a catastrophic complication after two-stage joint arthroplasty and there are multiple risk factors for the development of PJI after two-stage arthroplasty for SA, such as male sex and diabetes [54]. Physicians must clarify the risk factors through preoperative examination and discussion to minimise such risk factors. Due to the low detection rate of routine microbiological cultures, to clarify the type of microorganisms for targeted drug therapy, we recommend utilising mNGS for preoperative and intraoperative pathogen cultures to ensure complete control of infections prior to two-stage arthroplasty to reduce the risk of PJI.

### One-stage arthroplasty

While it is true that one-stage spacer implantation + two-stage arthroplasty has shown excellent results, there are still studies that indicate a high rate of complications after two-stage arthroplasty, mainly PJI. Tan et al. reported that the rate of postoperative PJI was 13.3% after two-stage arthroplasty following previous SA [54]. A study by Russo et al. showed that the complication rate after one-stage spacer implantation + two-stage arthroplasty was 20.2% [11].

The use of spacers increases the risk of complications such as spacer dislocation and spacer rupture. The results of a study by Anagnostakos et al. [15] showed that the rate of spacer-specific complications after two-stage arthroplasty was 23%, while the rate of spacer-nonspecific complications (mainly sinus tracts) was 50%. Spacer complications not only have a significant impact on postoperative recovery but also increase the risk of infection. Two-stage arthroplasty also suffers from the risks of multiple operations, high medical costs, long rehabilitation and antibiotic use time. Therefore, there is still a need to improve current surgical methods to further improve patient prognosis.

With improved pathogenetic testing and surgical techniques, the use of debridement in conjunction with one-stage arthroplasty for chronic PJI can achieve results similar to those of two-stage revision [57]. Compared with two-stage arthroplasty, one-stage arthroplasty has the advantages of a simpler surgical procedure, shorter antibiotic use, shorter hospitalisation and lower relative cost of treatment. Furthermore, the outcomes of one-stage arthroplasty are comparable to those of two-stage arthroplasty. Given that the current difficulty in the treatment of SHA lies in the clarification and eradication of causative organisms, it has been suggested that one-stage arthroplasty of SHAs is possible with clarification of the causative organism. Some studies have shown that one-stage arthroplasty has been successfully used for quiescent SHAs [22]. In addition, the findings of Wei et al. [52] suggest that delaying arthroplasty does not reduce the risk of PJI and that one-stage arthroplasty does not result in a greater rate of infection, providing theoretical support for the use of one-stage arthroplasty for chronic destructive SA.

Although there are few clinical studies, there are still studies that have shown satisfactory results with one-stage arthroplasty (Table 3). Papanna et al. [22] showed that one-stage arthroplasty had similar results in patients with SA and degenerative osteoarthritis (OA) compared to two-stage arthroplasty. In addition, one-stage arthroplasty also reduced the rate of postoperative complications. The study by Sultan et al. [14] included 8 patients who underwent one-stage hip arthroplasty, and the treatment success rate was 100%. A study by Tan et al. [53] showed that there was no significant difference in the efficacy or PJI rate between patients who underwent one-stage TJA and those who underwent two-stage TJA. Zhang et al. [7] compared the efficacy and PJI rate of pathogen + debridement + antibiotics + single-stage replacement (PDASR) versus two-stage arthroplasty for chronic destructive SHA. The results showed that there was no case of recurrence of infection in either group and there was no significant difference in the rates of readmission, reinfection, revision, dislocation, aseptic loosening, complications or HHS between the two groups. In addition, intraoperative blood loss, hospitalisation time and cost were significantly lower in the PDASR group compared to the two-stage arthroplasty group.

**Table 3:** Relevant studies of one-stage arthroplasty for SA

Literature	Distribution of main pathogens	Total detection rate	Surgical Cases	THA/TKA	Average follow-up (months)	Success rate
Zhang et al. [7], 2022	MSSA 9.1% (1/11), MRSA 9.1% (1/11), MSSE 9.1% (1/11)	100% (11/11)	11	THA	> 12	100%
Tan et al. [53], 2021	Staphylococcus aureus 24.2% (38/157)	67.5% (106/157)	97	THA&TKA	N/A	84.5%
Tan et al. [54], 2020	N/A	N/A	105	THA (31) &TKA (74)	62.4	88.6%
Sultan et al. [14], 2019	MSSA 25.8% (16/62), MRSA 4.8% (3/62)	61.3% (38/62)	8	THA	52.8	100%
Papanna et al. [22], 2018	N/A	N/A	7	THA	70	100%

*MRSA* Methicillin-resistant Staphylococcus Aureus, *MSSA* Methicillin-sensitive Staphylococcus Aureus, *N/A* Not Available, *SA* Septic Arthritis, *THA* Total Hip Arthroplasty, *TKA* Total Knee Arthroplasty

In a preliminary study, Zhang et al. [7] proposed a PDASR treatment plan, which is different from the common one-stage arthroplasty plan by focusing more on the identification of pathogens and sensitive drug treatment. Pus, synovial membrane or granulation tissue is obtained from the joint cavity before and during surgery to identify the pathogen. Sensitive antibiotics are used to fight infection to improve the success rate of subsequent surgery. In addition, the PDASR program pays extra attention to asepsis during debridement and arthroplasty. The risk of infection is minimised by the use of a surgical membrane, double gloves, changes in gowns and surgical instruments and copious irrigation. These practices, which are different from those of normal one-stage arthroplasty, help to minimise the occurrence of postoperative complications such as infections.

Despite the advantages of a simple surgical procedure, short duration of antibiotics and low cost of treatment, current treatment guidelines still consider active infection to be a relative contraindication to one-stage arthroplasty. Therefore, the indications for one-stage arthroplasty need to be strictly adhered to when selecting a regimen: the patient has a good nutritional status (normal haemoglobin and albumin levels), no history of immune dysfunction or previous multiple surgeries, a short duration of symptoms (< 12 weeks) and available microbiological data. There is a need for improved mNGS assays to further improve pathogen detection and reduce infection recurrence. With the general trend of increasing drug resistance in pathogenic bacteria, we also need to look for antibiotics with stronger anti-infective effects. It has been suggested in the literature that halicin (SU3327) remains active against Staphylococcus aureus in biofilms grown on orthopaedically relevant substrates [58]. Therefore, further animal model tests should be conducted to prove its effect.

C.Hamad et al. [59] argued that for a thorough treatment of PJI, surgical debridement must be comprehensive, and methods to visualise the microbiota must be developed to allow the removal of microscopic extensions. The advantages and limitations of various surgical treatments for SHA have been summarized in Table 4. The simultaneous development and use of adjunctive therapies beyond antimicrobial agents are necessary to achieve complete microbial eradication. This aligns with the concept of PDASR that we follow for the treatment of chronic destructive SHA. Additionally, Welling et al. [60] proposed that topical application of the hybrid bacterial tracer 99mTc-UBI29-41-Cy5 allows bacterial visualisation, therapy guidance and quantification of debridement effectiveness on femoral implants. These types of techniques may help us evaluate debridement and implantation outcomes in PDASR scenarios, reducing the probability of reinfection. Ji et al. [61] suggested that single-stage revision with intra-articular antibiotic infusion can provide high antibiotic concentrations in synovial fluid, thereby overcoming the reduced vascular supply and biofilm formation, which may be a viable option for treating PJI after multiple failed surgeries for reinfection. This study also provides important guidance for the use of antibiotics in one-stage arthroplasty for chronic destructive SHA.

**Table 4:** Advantages and limitations of various surgical treatments for SHA

Surgical treatment	Advantages	Limitations
Arthroscopic debridement	Less surgical trauma Faster functional recovery	Reinfection Multiple operations Acute infection only
Open debridement	Relatively good infection control	Reinfection Potential complication Acute infection only
Debridement + Girdlestone head and neck dissection	Complete infection removal	Activity limitation Potential complication
Debridement + Girdlestone femoral head and neck resection + two-stage arthroplasty	Complete infection removal	Activity limitation Potential complication
One-stage spacer therapy + two-stage arthroplasty	Limb length maintenance Function maintenance Less complication	Multiple operations Long-term antibiotic use and hospitalization High cost
One-stage arthroplasty	Simpler procedure Shorter antibiotic use and hospitalization Lower cost	Indication selection Available Microbiology

*SHA* Septic Hip Arthritis

Neufeld and E. F. Liechti et al. [62] independently suggested that the host and limb status according to the Musculoskeletal Infection Society (MSIS) staging system were not associated with subsequent infection-related failure in PJI patients. Whether these factors are risk factors for treatment failure in chronic destructive SHA remains unclear. However, more clinical trials are needed to confirm these findings.

Other postoperative complications are also important concerns. Kvarda et al. [63] proposed that patients who underwent revision surgery for PJI were at greater risk of perioperative myocardial injury (PMI) and death than those who underwent aseptic arthroplasty surgery. As an infectious disease, SHA may also pose such risks. Therefore, screening for PMI and treatment in specialised multidisciplinary units should be considered in SHA.

**Fig. 1:**
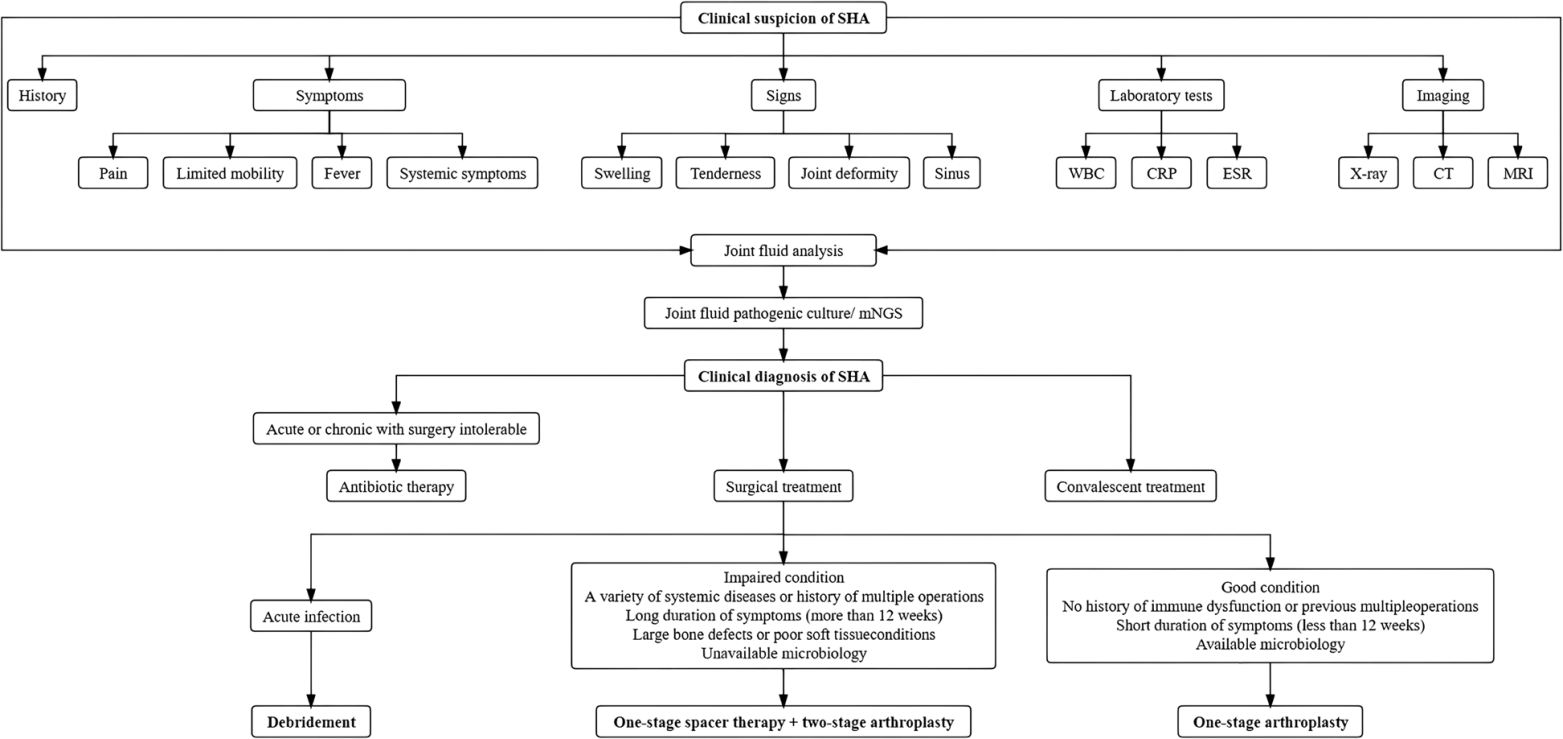
Clinical diagnosis and treatment of SHA

It has been suggested that adequate treatment of bone and joint infection (BJI) requires the consideration of multiple factors. Case discussion by a multidisciplinary team may enhance the management and study of patients with BJI and may reduce the rate of antimicrobial resistance [64]. A flowchart like Fig. 1 that sorts out disease diagnosis and treatment approaches can help us to further enhance the management of SHA.

Currently, most of the literature on one-stage arthroplasty for SHA consists of case‒control studies and retrospective cohort studies rather than randomised controlled trials (RCTs). The quality of the literature is poor, thus providing limited clinical evidence for us. In the case of one-stage arthroplasty for chronic destructive SHA, there is also a need for multicentre studies with better homogeneity, larger sample sizes and longer follow-up, with the joint efforts of multidisciplinary teams, to offer the possibility of applying this method in the clinic.

## Conclusion

In summary, there is still no consensus on the definition, diagnosis, or treatment of chronic destructive septic hip arthritis. Currently, one-stage spacer therapy + two-stage arthroplasty is considered the standard surgical treatment, with high success rates and low reinfection rates. However, this approach also has disadvantages, including a high incidence of interval spacer-related complications, multiple surgeries, high medical costs, long recovery times and prolonged antibiotic use. One-stage arthroplasty has emerged as a new surgical treatment option in recent years, offering advantages such as a simpler surgical procedure, shorter antibiotic use and lower treatment costs than two-stage arthroplasty. However, more high-quality research is needed to provide the latest evidence supporting the safety and feasibility of this treatment option. Regardless of the surgical approach adopted, the detection of pathogenic microorganisms remains paramount in the treatment of chronic destructive SHA. Novel molecular detection methods such as mNGS can assist traditional cultures by accurately identifying the infecting microorganisms, thereby aiding in disease treatment.

## Data Availability

Not required.

## Abbreviations

SHA: Septic hip arthritis
SA: Septic arthritis
PJI: Periprosthetic joint infection
CRP: C-reactive protein
ESR: Erythrocyte sedimentation rate
PMN: Polymorphonuclear leukocyte percentage
NLR: Neutrophil-to-lymphocyte ratio
SF-NLR: Synovial fluid NLR
MRI: Magnetic Resonance Imaging
NGS: Next-generation sequencing
mNGS: Metagenomic next-generation sequencing
MIC: Minimal inhibitory concentration
VAS: Visual analogue scale
MRSA: Methicillin-resistant Staphylococcus aureus
THA: Total hip arthroplasty
TJA: Total joint arthroplasty
OA: Osteoarthritis
PDASR: Pathogen + debridement + antibiotics + single-stage replacement
MSIS: Musculoskeletal Infection Society
PMI: Perioperative myocardial injury
BJI: Bone and joint infection
RCT: Randomised controlled trial
